# Conflict Adaptation in 5-Year-Old Preschool Children: Evidence From Emotional Contexts

**DOI:** 10.3389/fnhum.2019.00014

**Published:** 2019-01-29

**Authors:** Danfeng Li, Tongran Liu, Jiannong Shi

**Affiliations:** ^1^CAS Key Laboratory of Behavioral Science, Institute of Psychology, Chinese Academy of Sciences, Beijing, China; ^2^Department of Psychology, University of Chinese Academy of Sciences, Beijing, China; ^3^Department of Learning and Philosophy, Aalborg University, Aalborg, Denmark

**Keywords:** conflict adaptation, congruency sequence effects, facial expressions, preschool children, event-related potential

## Abstract

This research investigated the individual behavioral and electrophysiological differences during emotional conflict adaptation processes in preschool children. Thirty children (16 girls, mean age 5.44 ± 0.28 years) completed an emotional Flanker task (stimulus-stimulus cognitive control, S-S) and an emotional Simon task (stimulus-response cognitive control, S-R). Behaviorally, the 5-year-old preschool children exhibited reliable congruency sequence effects (CSEs) in the emotional contexts, with faster response times (RTs) and lower error rates in the incongruent trials preceded by an incongruent trial (iI trial) than in the incongruent trials preceded by a congruent trial (cI trial). Regarding electrophysiology, the children demonstrated longer N2 and P3 latencies in the incongruent trials than in the congruent trials during emotional conflict control processes. Importantly, the boys showed a reliable CSE of N2 amplitude when faced with fearful target expression. Moreover, 5-year-old children showed better emotional CSEs in response to happy targets than to fearful targets as demonstrated by the magnitude of CSEs in terms of the RT, error rate, N2 amplitude and P3 latency. In addition, the results demonstrated that 5-year-old children processed S-S emotional conflicts and S-R emotional conflicts differently and performed better on S-S emotional conflicts than on S-R emotional conflicts according to the comparison of the RT-CSE and P3 latency-CSE values. The current study provides insight into how emotionally salient stimuli affect cognitive processes among preschool children.

## Introduction

Early childhood is a key period for cognitive development (Rothbart et al., 2003; Rueda et al., 2004; Carlson, 2005). Children’s cognitive abilities in early childhood can predict their subsequent academic competence and their ability to cope with frustration and stress (Shoda et al., 1990; Clark et al., 2010; Allan and Lonigan, 2011; Bull et al., 2011). Early childhood is also essential for the development of emotional abilities, such as emotion expression, emotion understanding and emotion regulation (Carlson and Wang, 2007). Zelazo and Cunningham (2007) proposed the “interactive model” to explain the relationship between cognition and emotion and noted that emotion was never entirely independent of cognition and responded to the motivational aspect of cognition during conscious, goal-orientated problem solving. Etkin et al. (2006) used a face-word Stroop task to study the interaction between cognition and emotion processes. They asked participants to identify emotional facial expressions while ignoring emotional words; thus, an emotional conflict was generated when the emotional facial expression was incongruent with the emotional word, such as a happy expression with the word “fear.” Four emotional conflict conditions corresponded to the congruence between the preceding trial and the current trial: a congruent trial preceded by a congruent trial (cC condition), an incongruent trial preceded by a congruent trial (cI condition), a congruent trial preceded by an incongruent trial (iC condition), and an incongruent trial preceded by an incongruent trial (iI condition). Reliable emotional congruency sequence effects (CSEs) were indicated by faster response times (RTs) and lower error rates during the iI trials relative to those during the cI trials (Etkin et al., 2006), reflecting results comparable to those in non-emotional contexts (Gratton et al., 1992; Schmidt, 2013a). Although emotional CSEs have been observed in adults (Etkin et al., 2006; Chechko et al., 2014; Worsham et al., 2015), whether young children can show emotional CSEs remains unknown; therefore, in the present study, we explored the relationship between cognition and emotion in preschool children from the perspective of emotional CSEs.

CSEs have been reported to be related to conflict-monitoring theory (Botvinick et al., 2001; Kerns et al., 2004), feature-integration (Mayr et al., 2003; Hommel et al., 2004), contingency learning (Schmidt and De Houwer, 2011; Mordkoff, 2012), and temporal learning confounds (Schmidt, 2013a, b; Schmidt and Weissman, 2014). Conflict-monitoring theory posits that the levels of conflict in previous trials may improve performance in current trials by inducing top-down processes that increase cognitive control (Botvinick et al., 2001; Kerns et al., 2004). Contingency learning confounds are related to the presentation of a distractor with a congruent target more frequently than an incongruent target (Schmidt and De Houwer, 2011; Mordkoff, 2012). Feature repetition confounds are related to the repetition of a target and/or distractor in contiguous trials (Mayr et al., 2003; Hommel et al., 2004). Temporal learning confounds posit that learning is not only about selecting and executing the appropriate response but also when to respond (Schmidt, 2013a, b; Schmidt and Weissman, 2014).

The N2 and P3 components are two valid event-related potential (ERP) components used to evaluate reliable emotional CSEs (e.g., Clayson and Larson, 2013; Chechko et al., 2014). N2 is a negative deflection that peaks approximately 250–350 ms after stimulus presentation and originates in the anterior cingulate cortex (ACC); N2 is sensitive to emotional conflict monitoring (e.g., Chechko et al., 2014). P3 is related to emotional conflict resolution and the allocation of attentional control in the prefrontal cortex (PFC) and parietal areas (Clayson and Larson, 2013). Rueda et al. (2004) found a reliable parietal P3 conflict effect and a weak frontal N2 conflict effect in 4-year-old children, which indicated that 4-year-old children’s conflict effects may have a closer relation to the parietal P3 effect. Similarly, Davis et al. (2003) found no differences in the N2 amplitude between 6-year-old children and adults on the inhibition control task, and a late positive component (LPC, 550–600 ms) was observed in the children. CSEs in emotional contexts are reflected by lower amplitudes and shorter latencies of the N2 and P3 components in iI trials relative to those in cI trials. Furthermore, researchers often calculate the magnitude of CSE by the formula (cI − cC) − (iI − iC) (Nieuwenhuis et al., 2006). Larger (i.e., more positive) CSE magnitudes in terms of RTs, error rates, and P3 amplitudes and smaller (i.e., more negative) CSE magnitudes in terms of N2 amplitudes reflect inefficient conflict detection and resolution (Larson et al., 2012a). Larson et al. (2012a) found that 10-year-old children had similar CSEs compared to adults with respect to the conflict slow potential (SP) in a Stroop task. Liu et al. (2018) further indicated that 5-year-old children had reliable behavioral CSEs on both Flanker and Simon tasks and showed reliable CSEs in N2 amplitude and P3 latency only in the Simon task (Liu et al., 2018). These results suggested that young children can show reliable CSEs in non-emotional contexts, and their performance was related to neural activation of both parietal and frontal areas. The current study investigated whether young children had reliable CSEs in an emotional context, and whether the emotional CSEs were due to frontal activation (N2 responses) or parietal activation (P3 responses).

The face-word Stroop task is not suitable for preschool children because it requires word recognition, and young children cannot recognize words describing facial expressions. To solve this problem, we used the emotional Flanker task and the emotional Simon task to explore CSEs in the present study. The emotional Flanker and Simon tasks are valid, graphical and do not require word recognition ability. In the emotional Flanker task (Liu et al., 2013), five facial expressions (fearful or happy) are shown in a horizontal row, with a central target facial expression and four bilateral facial expressions (the two on each side of the central target). An emotional conflict occurs when the target expression is incongruent with the distractor expressions. In the emotional Simon task (Xue et al., 2016), either a happy or a fearful expression is presented on the left or right side of a central fixation point, and participants are asked to identify the emotional facial expression by responding with their left or right hand (e.g., left hand for fearful expression and right hand for happy expression) while ignoring the location of the presented facial stimulus. Emotional conflicts are generated from incongruence between the location of the presented facial expression and the response hand. According to the dimensional overlap theory (Kornblum et al., 1990, 1999), conflicts can be categorized into different types by the overlap between the task-relevant stimulus (S_R_), the task-irrelevant stimulus (S_I_), and the response (R). Thus, the Flanker task belongs to the stimulus-stimulus (S-S) conflict type, where S_R_ (the direction of the target stimuli) overlaps with S_I_ (the direction of the distractor stimuli), while the Simon task belongs to the stimulus-response (S-R) conflict type, where S_I_ (the location of a presented stimuli) overlaps with R (the response hand). In non-emotional contexts, Jongen and Jonkman (2008) found that children exhibited different processing of S and R interferences, suggesting that S interference control reached mature levels (6–7-year-old children) earlier than R interference control (continued until early adolescence: 10–12-year-old children). Five-year-old children have been shown to exhibit varying performance between non-emotional S-S and S-R CSEs, with faster responses, higher accuracy and shorter N2 and P3 latencies in the S-S conflict task relative to those in the S-R task, possibly because 5-year-old children experienced a substantially greater cognitive load when they completed the S-R tasks vs. the S-S tasks in the non-emotional context (Liu et al., 2018). The question of whether S-S and S-R tasks have different substrates in the emotional contexts for young children is still unknown. Therefore, in the present research, we employed the emotional Flanker task and the emotional Simon task to compare differences in emotional CSEs for S-S and S-R conflicts in 5-year-old preschool children.

Some previous studies in adults have confirmed that specific target expressions (fearful and happy) can affect CSEs in emotional contexts. For example, Chechko et al. (2014) found that adults had faster RTs when faced with happy target expressions than when faced with fearful target expressions. Consistent with these results, Padmala et al. (2011) illustrated that negative emotion impaired CSEs in adult samples. Specifically, CSEs decreased when a negative stimulus was delivered between the main conflict stimuli. These results may be explained by the fact that according to the dual competition model, the cognitive control resources that are needed in conflict processing are shared by the processing of negative images (Pessoa, 2009). Additionally, the ability to process emotional stimuli substantially improves during early childhood. Batty and Taylor (2006) found that 4- to 5-year-olds have a longer evoked P1 latency (sensitive to face processing) when presented with fearful faces relative to happy faces, and boys had longer P1 latencies than girls, indicating that girls perform better than boys in facial processing. Boyatzis et al. (1993) also found that girls at 5 years of age performed significantly better than boys in emotion identification. However, how these specific target expressions (fear and happy) affect CSEs in preschool children remains unknown. Therefore, the current study also explored how different emotional valences affected the interaction between emotions and CSEs.

The main aim of this study was to explore the behavioral and electrophysiological performance of emotional CSEs in 5-year-old preschool children using the emotional Flanker (S-S) task and the emotional Simon (S-R) task. We selected 5-year-old children for the following reasons: first, cognition and emotion develop quickly in 3- to 5-year-olds (Rueda et al., 2004; Carlson, 2005; Carlson and Wang, 2007). Second, a previous study showed that even by 5 years old, the ability to correctly discriminate a range of emotional expressions is only rudimentary (Nelson, 1987); thus, at 5 years of age, children may be at the beginning stages of both cognitive and emotional development. We hypothesized the following: (1) based on previous results showing that 5-year-old children display reliable behavioral CSEs in non-emotional contexts and rapidly develop emotion ability (Carlson and Wang, 2007; Liu et al., 2018), we posited that 5-year-old children would exhibit behavioral CSEs in emotional contexts with faster RTs and lower error rates on iI trials than on cI trials. For electrophysiological CSEs, reliable emotional CSEs in children would be primarily related to the P3 component but not the N2 component; (2) similar to previous non-emotional CSE studies involving 4- to 5-year-old children (Rueda et al., 2004; Liu et al., 2018), children would show distinctive processes in S-S and S-R emotional tasks; and (3) as 5-year-olds exhibit better facial expression processing of happy faces than fearful faces (Batty and Taylor, 2006), we hypothesized that emotional CSEs may be modulated by target facial expressions, and that 5-year-old children may show better performance when presented with happy target expressions rather than fearful target expressions.

## Materials and Methods

### Ethics Statements

This study was carried out in accordance with the recommendations of the Institute of Psychology, Chinese Academy of Sciences, and written informed consent was obtained from each participant’s parents in accordance with the Declaration of Helsinki. The protocol was approved by the Institute of Psychology, Chinese Academy of Sciences.

### Participants

All participants were recruited from a community kindergarten. Participants in the final sample consisted of 30 children (*M* = 5.44 years, SD = 0.28; 16 girls). According to a questionnaire completed by their parents, all participants were right-handed, born full-term, and free from clinical disorders or uncorrected visual impairments. The parents provided written informed consent before participation of their child.

### Materials and Procedure

**Figure 1:**
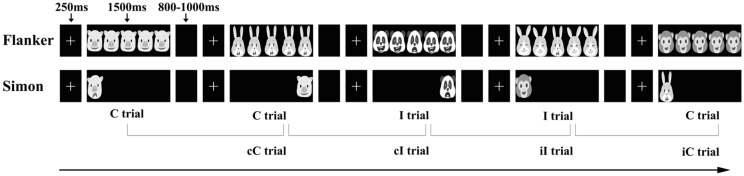
Representative examples of the emotional Flanker task and emotional Simon task. Note: C, congruent trial; I, incongruent trial; cC, congruent trial preceded by a congruent trial; cI, incongruent trial preceded by a congruent trial; iC, congruent trial preceded by an incongruent trial; iI, incongruent trial preceded by an incongruent trial.

We used the child-friendly emotional Flanker and Simon tasks to measure emotional CSEs. Stimuli were presented with E-prime software (Psychology Software Tools, Inc., Sharpsburg, PA, USA) and consisted of three male cartoon characters and three female cartoon characters, with one happy and one fearful expression for each cartoon character. Moreover, all the cartoon expressions had similar luminance. Prior to the formal experiment, 10 preschool children (mean age: 5.21 ± 0.15 years) were asked to provide valence ratings, or specifically, to judge the facial expression of the cartoon characters as happy or fearful, and the accuracy rate was 100%. In the formal experiment, the participants were tested individually in a comfortably seated position in a sound-damped and electrically shielded room. Stimuli were presented on a 17-inch Dell computer monitor positioned approximately 50 cm from the participants. Each task consisted of 276 trials (16 trials for the practice block and 260 trials for four formal experimental blocks). Each trial began with a central fixation “+” for 250 ms, followed by the stimulus presentation (1,500 ms). If participants took longer than 1,500 ms to respond, the trial was marked as an error of omission. The intertrial interval (ITI) was randomly varied between 800 and 1,000 ms. The stimuli used in the task were presented in a pseudorandom order to eliminate confounding effects due to the repetitions of the same face as the target and/or distracter across trials (Hommel et al., 2004) and counterbalanced across the trial types of expression, response button and gender. The proportions of congruent and incongruent trials were 50% each. These two tasks lasted for approximately 45 min. The participants were given a 2- to 3-min break after each block and a 5-min break between the two tasks. The stimuli and procedures for the two tasks are displayed in Figure 1.

#### Emotional Flanker Task

A modified child-friendly emotional Flanker task was used to measure S-S CSEs. In this task, each stimulus contained five faces of one identical model on a horizontal row, with a central target facial expression and four distractor facial expressions (two on each side). The visual angles of the stimulus were 4° vertically and 7° horizontally. Based on the congruency between the target and distractor expressions, the stimuli formed two conditions: a congruent condition (the target expression and distractor expressions were the same) and an incongruent condition (the target expression was different from the distractor expressions), as shown in Figure 1. The participants were instructed to judge the central target facial expressions and to ignore the distractor expressions by pressing the left response button or the right button. The mappings between response buttons and expressions were balanced among the participants.

#### Emotional Simon Task

A modified child-friendly emotional Simon task was used to measure S-R CSEs. In each trial, participants observed either a happy or a fearful expression on the left or right side of their visual field. The visual angles of the stimuli were 3° vertical and 3° horizontal. The participants were asked to identify the expression using the left or right button while ignoring the location of the facial expression (left or right). Based on the stimulus location and response button, two conditions existed: a congruent condition (the presentation location and the response button were ipsilateral) or an incongruent condition (the presentation location and the response button were contralateral).

### Electroencephalogram Recording and Processing

Electroencephalogram (EEG) data were recorded from 32 scalp sites using a SynAmps 2 amplifier system (Neuroscan USA Ltd.; DC, on-line bandpass = 0.10–100 Hz) with an extended 10–20 system for electrode locations. Horizontal and vertical electrooculograms (EOGs) were recorded bipolarly with the electrodes placed at the outer canthi of both eyes and above and below the left eye. Impedances were maintained below 5 kΩ. The EEG signal was continuously recorded at a sample rate of 1,000 Hz using the nose reference. The EEG was epoched with −100 ms prior to and 1,000 ms after the onset of the stimuli, and the time window of −100 ms to 0 ms was submitted to baseline correction. The off-line filter was at 0.1–30 Hz, and epochs exceeding a threshold of ±100 μV were removed from further analyses. After artifact rejection, 43.35 ± 448 cC trials, 37.45 ± 5.02 cI trials, 37.79 ± 4.71 iC trials, and 40.2 ± 4.83 iI trials were carried out in the emotional Flanker task, and 41.46 ± 4.6 cC trials, 36.71 ± 4.33 cI trials, 36.57 ± 4.7 iC trials, and 39.39 ± 5.08 iI trials were carried out in the emotional Simon task.

According to previous studies (Rueda et al., 2004) and the current data, the electrophysiological analysis of the N2 component was focused on a time window of 300–550 ms (average for the electrodes at F3, Fz, F4, FC3, FCz, and FC4), while the P3 component was 500 ms to 700 ms (average for the electrodes at CP3, CPZ, CP4, P3, PZ, and P4). The N2 and P3 mean amplitudes were derived using the adaptive mean procedure (Larson et al., 2012a,b) in which the peak amplitude was first identified, and then an average was computed for the 15 ms before to the 15 ms after the peak amplitude.

### Data Analysis

Behavioral and ERP data were analyzed using SPSS 21.0 (IBM Inc., NY, USA). Error trials and the first trials for each block were excluded from further analyses. Two ANOVAs with Bonferroni multiple comparison tests were used in this study. For these analyses, Bonferroni multiple testing correction was set at *p* ≤ 0.025 (*p* = 0.05 divided by two, i.e., the number of tests used), which is consistent with a previous study (Li et al., 2017).

The first ANOVAs were conducted to determine whether 5-year-old children exhibited reliable emotional CSEs, with shorter RTs and lower error rates in iI trials compared with cI trials, as well as more negative N2 amplitudes, more positive P3 amplitudes, longer N2 latencies, and longer P3 latencies in cI trials compared with iI trials. The mean RTs, error rates, peak latencies and mean amplitudes of N2 and P3 were analyzed with target expression (fearful or happy), task type (emotional Flanker task or emotional Simon task), previous trial (congruent or incongruent) and current trial (congruent or incongruent) as the within-subject variables and gender (boy or girl) as the between-subjects variable. The second ANOVAs were conducted to explore the magnitude of emotional CSEs and identify specific gender-related, target expression-related and task type-related differences in emotional CSEs among 5-year-old children. The magnitudes of CSEs were calculated by the formula (cI − cC) − (iI − iC) (Nieuwenhuis et al., 2006); larger (i.e., more positive) CSE magnitudes in terms of RTs, error rates, and P3 amplitudes, and smaller (i.e., more negative) CSE magnitudes in terms of N2 amplitudes reflect inefficient conflict detection and resolution (Larson et al., 2012b). The magnitudes of CSEs (RT-CSE values, error rate-CSE values, N2-CSE values and P3-CSE values) were subjected to 2 × 2 × 2 ANOVAs. Target expression (fearful, happy) and task type (emotional Flanker task, emotional Simon task) were the within-subject factors, and gender (boy, girl) was the between-subjects factor. Finally, a correlation analysis was conducted between the behavioral (RT-CSE values and ACC-CSE values) and ERP data (mean amplitude-CSE values, peak latencies-CSE values).

## Results

### Behavioral Results

Table 1 shows the mean RTs and error rates of the 5-year-old children for the emotional Flanker and Simon tasks, and the results of the ANOVA analyses of RTs and error rates are presented in Table 2 (For the congruency effects, please see the Supplementary Materials).

**Table 1:** The mean reaction time (ms), error rates and magnitudes of emotional CSEs in 5-year-old children.

		Flanker		Simon	
		Fearful	Happy	Fearful	Happy
cC	RT	948.83 (129.20)	929.55 (129.62)	1028.39 (100.70)	965.80 (98.01)
	Error rate	0.18 (0.12)	0.19 (0.13)	0.22 (0.13)	0.21 (0.13)
cI	RT	996.29 (136.32)	983.06 (137.72)	1091.22 (82.17)	1065.01 (88.89)
	Error rate	0.30 (0.11)	0.32 (0.13)	0.29 (0.16)	0.29 (0.14)
iC	RT	1009.87 (121.39)	922.48 (129.67)	1088.90 (92.91)	1045.58 (99.70)
	Error rate	0.33 (0.15)	0.26 (0.11)	0.31 (0.18)	0.29 (0.15)
iI	RT	983.95 (130.27)	954.96 (159.64)	1047.21 (92.51)	1002.14 (89.66)
	Error rate	0.22 (0.10)	0.26 (0.13)	0.27 (0.14)	0.21 (0.12)
CSEs	RT	73.39 (93.87)	21.02 (122.01)	114.53 (103.17)	108.65 (93.19)
	Error rate	0.22 (0.16)	0.13 (0.18)	0.20 (0.17)	0.18 (0.18)

*Note: cC, congruent trial preceded by a congruent trial; cI, incongruent trial preceded by a congruent trial; iC, congruent trial preceded by an incongruent trial; iI, incongruent trial preceded by an incongruent trial. CSEs, congruency sequence effects*.

**Table 2:** Results of the ANOVAs of reaction time and error rates in 5-year-old children.

	RT			Error rate		
	*F*	*p*	*η*^2^	*F*	*p*	*η*^2^
T	19.79	<0.001	0.41	0.92	>0.05	0.03
E	32.36	<0.001	0.54	6.98	<0.01	0.20
P	2.90	<0.001	0.10	5.04	<0.03	0.15
C	18.41	<0.001	0.40	4.85	<0.03	0.15
G	0.29	>0.05	0.01	7.63	<0.01	0.21
T × G	0.03	>0.05	0.00	2.25	>0.05	0.03
E × G	0.08	>0.05	0.00	1.86	>0.05	0.06
C × G	0.21	>0.05	0.01	0.48	>0.05	0.02
T × E	0.41	>0.05	0.02	4.68	<0.04	0.14
T × C	0.29	>0.05	0.01	1.63	>0.05	0.06
E × C	9.51	<0.01	0.25	5.02	<0.03	0.15
P × C	44.46	<0.001	0.61	98.85	<0.001	0.78
T × E × G	0.18	>0.05	0.01	0.01	>0.05	0.00
T × C × G	0.09	>0.05	0.00	0.07	>0.05	0.00
E × C × G	1.48	>0.05	0.05	3.89	>0.05	0.12
T × E × C	1.42	>0.05	0.05	10.95	<0.001	0.28
P × C × G	0.26	>0.05	0.01	2.43	>0.05	0.08
T × P × C	15.17	<0.001	0.35	0.05	>0.05	0.01
E × P × C	0.19	>0.05	0.01	4.59	<0.04	0.14
T × E × C × G	6.42	<0.02	0.19	0.43	>0.05	0.02
T × P × C × G	0.54	>0.05	0.02	6.64	<0.02	0.19
E × P × C × G	0.09	>0.05	0.00	1.58	>0.05	0.05
T × E × P × C	8.74	<0.01	0.24	1.13	>0.05	0.04
T × E × P × C × G	2.49	>0.05	0.08	1.49	>0.05	0.05

*Note: T, task type; E, expression; P, previous trial; C, current trial; G, gender*.

### RTs

Regarding the mean RTs, the previous trial × current trial interaction was significant, *F*_(1,28)_ = 5.74, *p* < 0.05, *η*^2^ = 0.17. The *post hoc* Bonferroni tests reflected that the RTs were shorter in the iI trials than the cI trials (*t*_(29)_ = 6.20, *p* < 0.001) and shorter in the cC trials than the iC trials (*t*_(29)_ = 5.40, *p* < 0.001). A significant four-way interaction was found for previous trial × current trial × target expression × task type, *F*_(1,28)_ = 8.74, *p* < 0.01, *η*^2^ = 0.24. The Bonferroni *post hoc* tests showed that in the emotional Flanker task, the children had shorter RTs in the cC trials than the iC trials when faced with a fearful target expression (*t*_(29)_ = 4.88, *p* < 0.001) and shorter RTs in the iI trials than the cI trials when faced with a happy target expression (*t*_(29)_ = 2.23, *p* = 0.024), whereas in the emotional Simon task, the children responded more quickly in the iI trials than the cI trials and had shorter RTs in the cC trials than the iC trials in response to both fearful and happy target expressions (fearful: *t*_(29)_ = 3.84, *p* < 0.001, *t*_(29)_ = 4.78, *p* = 0.001; happy: *t*_(29)_ = 4.57, *p* < 0.001, *t*_(29)_ = 6.43, *p* < 0.001).

Regarding the RT-CSE values, a significant main effect of task type was found, *F*_(1,28)_ = 15.17, *p* = 0.001, *η*^2^ = 0.35, as smaller RT-CSE values were observed in the emotional Flanker task than the emotional Simon task, *t*_(29)_ = 3.89, *p* = 0.001. The interaction between the target expression and task type was significant, *F*_(1,28)_ = 8.74, *p* = 0.006, *η*^2^ = 0.24. The Bonferroni multiple comparison tests revealed that when faced with happy target expressions, the children displayed smaller RT-CSE values in the emotional Flanker task than the emotional Simon task, *t*_(29)_ = 4.60, *p* < 0.001. In the emotional Flanker task, the children displayed smaller RT-CSE values when faced with the happy expressions than when faced with fearful expressions, *t*_(29)_ = 2.31, *p* = 0.019. The main effect of target expression was not significant, *F*_(1,28)_ = 0.19, *p* > 0.05, *η*^2^ = 0.01. There was no significant main effect of gender, *F*_(1,28)_ = 0.26, *p* > 0.05, *η*^2^ = 0.01. The other interactions were not significant, *p*s > 0.05.

### Error Rates

Regarding the mean error rates, the previous trial × current trial interaction was significant, *F*_(1,28)_ = 98.85, *p* < 0.001, *η*^2^ = 0.78. The Bonferroni *post hoc* tests showed that the children had higher error rates in the cI trials than the iI trials (*t*_(29)_ = 7.00, *p* < 0.001) and in the iC trials than the cC trials (*t*_(29)_ = 9.08, *p* < 0.001).

Regarding the error rate-CSE values, the main effect of target expression was significant, *F*_(1,28)_ = 4.59, *p* < 0.05, *η*^2^ = 0.14, as the children had smaller error rate-CSE values when faced with happy expressions than when faced with fearful expressions, *t*_(29)_ = 2.17, *p* < 0.05. Moreover, the interaction of task type and gender was significant, *F*_(1,28)_ = 6.64, *p* < 0.05, *η*^2^ = 0.19. The corrected Bonferroni *post hoc* tests showed that the girls displayed smaller error rate-CSE values than the boys in the emotional Flanker task, *t*_(29)_ = 3.17, *p* < 0.005. No significant main effect of task type was found, *F*_(1,28)_ = 0.05, *p* > 0.05, *η*^2^ = 0.01, and no significant main effect of gender was observed, *F*_(1,28)_ = 2.43, *p* > 0.05, *η*^2^ = 0.08. The other interactions were not significant, *p*s > 0.05.

### ERP Results

**Figure 2:**
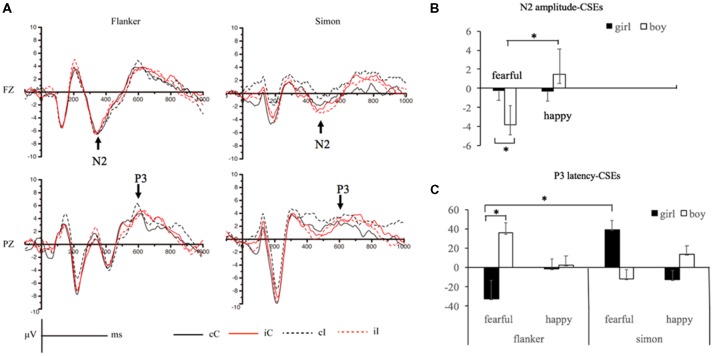
Panel **(A)** shows the averaged N2 and P3 waveforms in the Flanker and Simon tasks, respectively. Panel **(B)** Presents the congruency sequence effects (CSEs) of N2 amplitude. Panel **(C)** shows the CSEs of P3 latency. *Note*: cC, congruent trial preceded by a congruent trial; cI, incongruent trial preceded by a congruent trial; iC, congruent trial preceded by an incongruent trial; iI, incongruent trial preceded by an incongruent trial. *indicates *p* < 0.05.

The means and standard deviations of the amplitudes, latencies and CSEs of the N2 and P3 components among the 5-year-old children are presented in Table 3, and the ANOVAs of the N2 and P3 amplitudes and latencies are shown in Table 4. The grand average waveforms of the N2 and P3 components are displayed in Figure 2 (For the congruency effects, please see the Supplementary Materials).

**Table 3:** The mean amplitudes and peak latencies of N2 and P3 and the magnitude of emotional CSEs in 5-year-old children.

		Flanker		Simon	
		Fearful	Happy	Fearful	Happy
N2 amplitude	cC	−7.63 (4.69)	−8.66 (5.46)	−5.88 (4.87)	−6.10 (4.36)
	cI	−9.23 (5.31)	−8.56 (4.74)	−7.47 (5.04)	−4.21 (5.58)
	iC	−8.20 (4.75)	−8.80 (4.76)	−7.37 (5.43)	−7.42 (4.85)
	iI	−7.29 (4.86)	−8.77 (3.42)	−7.61 (5.68)	−6.53 (3.49)
	CSEs	−2.52 (9.83)	0.07 (8.03)	−1.35 (7.12)	1.00 (5.63)
N2 latency	cC	390.95 (56.10)	364.06(40.17)	426.79(67.36)	432.13(64.89)
	cI	396.56 (49.53)	384.53(54.19)	444.21(72.17)	437.82(82.19)
	iC	381.32 (55.12)	388.07(63.44)	446.39(73.80)	440.67(66.87)
	iI	388.04 (54.79)	401.54(59.57)	455.48(60.68)	450.81(70.24)
	CSEs	−1.12 (80.98)	7.01 (97.40)	8.33 (87.86)	−4.45 (98.48)
P3 amplitude	cC	8.07 (6.28)	8.77 (7.13)	6.74 (5.60)	7.97 (5.42)
	cI	7.68 (6.53)	9.67 (6.03)	6.17 (5.73)	10.11 (8.29)
	iC	8.18 (4.94)	8.91 (5.30)	7.48 (6.08)	7.50 (5.07)
	iI	8.43 (6.49)	8.59 (5.78)	7.08 (4.06)	8.65 (5.18)
	CSEs	−0.65 (8.21)	1.22 (8.82)	−0.18 (7.96)	0.99 (9.61)
P3 latency	cC	609.19 (48.66)	598.29(44.76)	590.68(59.55)	623.26(54.73)
	cI	619.11 (48.54)	609.96(42.82)	611.03(49.64)	618.05(65.84)
	iC	603.03 (53.43)	604.88(46.99)	616.37(61.35)	617.87(53.92)
	iI	613.76 (48.41)	616.05(53.70)	621.36(58.61)	613.17(50.51)
	CSEs	−0.82 (92.46)	0.48 (99.29)	15.37 (90.48)	−0.51 (77.15)

*Note: cC, congruent trial preceded by a congruent trial; cI, incongruent trial preceded by a congruent trial; iC, congruent trial preceded by an incongruent trial; iI, incongruent trial preceded by an incongruent trial. CSEs, congruency sequence effects*.

**Table 4:** Results of the ANOVAs of the N2 and P3 mean amplitudes and latencies in 5-year-old children.

	N2						P3					
	Mean amplitude			Peak latency			Mean amplitude			Peak latency		
	*F*	*p*	*η*^2^	*F*	*p*	*η*^2^	*F*	*p*	*η*^2^	*F*	*p*	*η*^2^
T	7.02	<0.01	0.20	32.28	<0.001	0.54	1.20	>0.05	0.04	0.36	>0.05	0.01
E	0.58	>0.05	0.02	0.51	>0.05	0.03	9.59	<0.001	0.26	0.39	>0.05	0.01
P	1.99	>0.05	0.07	5.85	<0.02	0.17	0.01	>0.05	0.01	0.68	>0.05	0.02
C	0.04	>0.05	0.01	3.48	<0.04	0.11	0.85	>0.05	0.03	4.02	<0.04	0.13
G	0.20	>0.05	0.01	0.04	>0.05	0.01	0.12	>0.05	0.01	0.89	>0.05	0.03
T × G	2.29	>0.05	0.08	0.98	>0.05	0.03	6.18	<0.02	0.18	0.10	>0.05	0.01
E × G	2.82	>0.05	0.09	0.06	>0.05	0.01	0.36	>0.05	0.01	0.63	>0.05	0.02
C × G	0.16	>0.05	0.01	1.46	>0.05	0.05	0.27	>0.05	0.01	0.06	>0.05	0.00
T × E	6.38	<0.02	0.19	0.05	>0.05	0.00	1.01	>0.05	0.04	2.55	>0.05	0.08
T × C	0.28	>0.05	0.01	0.01	>0.05	0.00	0.56	>0.05	0.02	1.01	>0.05	0.04
E × C	2.15	>0.05	0.07	0.14	>0.05	0.01	1.80	>0.05	0.06	0.61	>0.05	0.02
P × C	1.02	>0.05	0.04	0.17	>0.05	0.01	0.26	>0.05	0.01	0.21	>0.05	0.01
T × E × G	0.59	>0.05	0.02	0.39	>0.05	0.01	0.02	>0.05	0.01	0.11	>0.05	0.01
T × C × G	0.22	>0.05	0.01	0.05	>0.05	0.01	2.99	>0.05	0.10	0.78	>0.05	0.03
E × C × G	1.75	>0.05	0.06	1.77	>0.05	0.06	0.05	>0.05	0.01	0.38	>0.05	0.01
E × P × C	3.84	>0.05	0.12	0.01	>0.05	0.04	0.86	>0.05	0.03	0.23	>0.05	0.01
T × E × C	0.90	>0.05	0.03	0.52	>0.05	0.02	1.57	>0.05	0.05	1.19	>0.05	0.04
P × C × G	0.38	>0.05	0.01	0.27	>0.05	0.01	0.32	>0.05	0.01	0.44	>0.05	0.02
T × P × C	0.44	>0.05	0.02	0.02	>0.05	0.00	0.01	>0.05	0.00	0.12	>0.05	0.01
T × E × C × G	0.45	>0.05	0.02	0.45	>0.05	0.02	7.78	<0.001	0.22	0.75	>0.05	0.03
T × P × C × G	0.08	>0.05	0.01	1.51	>0.05	0.02	0.02	>0.05	0.01	2.00	>0.05	0.07
E × P × C × G	3.98	<0.04	0.12	0.59	>0.05	0.02	0.21	>0.05	0.01	0.04	>0.05	0.01
T × E × P × C	0.14	>0.05	0.00	0.25	>0.05	0.01	0.02	>0.05	0.01	0.16	>0.05	0.01
T × E × P × C × G	0.15	>0.05	0.01	0.19	>0.05	0.01	0.99	>0.05	0.03	5.29	<0.02	0.16

*Note: T, task type; E, expression; P, previous trial; C, current trial; G, gender*.

### N2 Responses

Regarding the N2 mean amplitudes, the previous trial × current trial × target expression × gender interaction was significant, *F*_(1,28)_ = 3.98, *p* < 0.05, *η*^2^ = 0.12, and the corrected multiple comparison tests showed that when faced with fearful target expressions, the boys showed more negative N2 amplitudes in the cI trials than the iI trials, *t*_(29)_ = 2.31, *p* < 0.02.

Regarding the N2 amplitude-CSE values, there was a significant main effect of target expression, *F*_(1,28)_ = 3.84, *p* < 0.05, *η*^2^ = 0.12. The N2 amplitude-CSE values in response to the happy target expressions were larger than those in response to the fearful target expressions, *t*_(29)_ = 1.96, *p* < 0.05. There was a significant two-way interaction between target expression and gender, *F*_(1,28)_ = 3.98, *p* < 0.05, *η*^2^ = 0.12. The corrected Bonferroni multiple comparison tests revealed that when faced with fearful target expressions, the girls had larger N2 amplitude-CSE values than the boys, *t*_(28)_ = 1.88, *p* < 0.02; the boys showed larger N2 amplitude-CSE values when faced with happy target expressions than when faced with fearful target expressions, *t*_(13)_ = 2.76, *p* < 0.01. The other interactions were not significant, *p*s > 0.05.

Regarding N2 latency, no significant interaction effects of “previous trial × current trial” were found, and no significant main effects or interaction effects were found for N2 latency-CSE values.

### P3 Responses

For P3 amplitudes, no significant interaction effects of “previous trial × current trial” were found, and no significant main effects or interaction effects were found for P3 amplitude-CSE values.

Regarding the means of the P3 latencies, there was a significant previous trial × current trial × target expression × gender × task type interaction, *F*_(1,28)_ = 5.29, *p* < 0.05, *η*^2^ = 0.16. The corrected Bonferroni *post hoc* tests showed that when faced with fearful target expressions, the girls exhibited longer P3 latencies in the iC trials than the cC trials in the emotional Simon task, *t*_(15)_ = 2.24, *p* < 0.02.

Regarding the P3 latency-CSE values, The interaction of target expression, task type and gender was significant, *F*_(1,28)_ = 5.29, *p* < 0.05, *η*^2^ = 0.16. The corrected Bonferroni *post hoc* tests showed that when the girls were faced with fearful target expressions, they displayed smaller P3 latency-CSE values in the emotional Flanker task than in the emotional Simon task, *t*_(15)_ = 2.77, *p* < 0.01, but when faced with fearful target expressions, the girls exhibited smaller P3 latency-CSE values than the boys, *t*_(28)_ = 2.14, *p* < 0.02, in the emotional Flanker task. The other interactions were not significant, *p*s > 0.05. No significant main effects of task type, *F*_(1,28)_ = 0.12, *p* > 0.05, *η*^2^ = 0.01, gender, *F*_(1,28)_ = 0.44, *p* > 0.05, *η*^2^ = 0.02, or target expression, *F*_(1,28)_ = 0.23, *p* > 0.05, *η*^2^ = 0.01, were found.

### Correlations Between Behavioral and ERP Data

The correlations between the behavioral and ERP data among the 5-year-old children are presented in Table 5. On the emotional Flanker task, the RT-CSE values and N2 latency-CSE values were significantly positively correlated, *r* = 0.41, *p* < 0.05. On the emotional Simon task, the error rate-CSE values were significantly positively correlated with the P3 amplitude-CSE values, *r* = 0.35, *p* < 0.05.

**Table 5:** Correlations between the CSEs of behavioral and ERP data in 5-year-old children.

	1	2	3	4	5	6	7	8	9	10	11	12
1. F_RT	−											
2. F_Error	0.24	−										
3. F_N2 amplitude	**−0.01**	**−0.08**	−									
4. F_N2 latency	0.41*	**0.12**	0.44*	−								
5. F_P3 amplitude	**−0.18**	**0.17**	0.62**	0.24	−							
6. F_P3 latency	**−0.08**	**0.20**	−0.05	0.29	0.09	−						
7. S_RT	0.26	0.43*	−0.14	−0.02	0.20	0.10	−					
8. S_Error	0.18	0.23	0.27	0.16	0.14	0.29	0.15	−				
9. S_N2 amplitude	−0.17	0.01	−0.06	−0.24	0.16	−0.22	**0.24**	**0.09**	−			
10. S_N2 latency	0.05	0.04	−0.23	−0.27	−0.29	−0.15	**0.02**	**0.03**	0.38*	−		
11. S_P3 amplitude	−0.29	0.12	−0.11	0.05	0.01	0.23	**0.14**	0.35*	0.18	−0.14	−	
12. S_P3 latency	−0.10	−0.15	−0.42*	−0.25	−0.51*	0.01	**0.01**	**−0.14**	0.20	0.46*	0.31	−

Note: *p < 0.05; **p < 0.01; F, Flanker task; S, Simon task. The most important correlations were in bold type and underlined in the table.

## Discussion

The current study explored the relationship between cognition and emotion in 5-year-old children using child-friendly emotional Flanker (S-S) and Simon (S-R) tasks. As expected, the 5-year-old children showed reliable emotional CSEs in behavioral (RT and error rate) and physiological (N2 amplitude, only boys) indices. Moreover, they showed better behavioral and electrophysiological processes on S-S emotional conflicts than on S-R emotional conflicts according to the RT-CSE values and P3 latency-CSE values. The current study also revealed some interesting results regarding the effects of target expressions during emotional CSEs, namely, the 5-year-old children’s identification of happy target expressions was better than their identification of fearful expressions in the emotional CSE context.

### Reliable Emotional CSEs in Young Children

Some previous studies have used Flanker or Simon tasks to explore CSEs in preschool children, but most of these studies focused on non-emotional contexts (e.g., Blair et al., 2004; Rueda et al., 2004; Carlson, 2005; Röthlisberger et al., 2010). The present study further found that 5-year-old preschool children displayed reliable emotional CSEs in both the emotional Flanker and Simon tasks, with faster RTs and lower error rates in the iI trials than in the cI trials, suggesting that 5-year-old children can adjust their cognitive control in current trials based on previous trials. These results are consistent with those of previous studies focusing on non-emotional CSEs in participants of the same age (Liu et al., 2018). Liu et al. (2018) explored developmental changes in CSEs among 5-year-old children, 10-year-old children and young adults in non-emotional contexts. They found that similar to older children and young adults, 5-year-old children showed reliable non-emotional CSEs according to RTs and error rates.

Moreover, the electrophysiological findings revealed that the 5-year-old preschool children had reliable emotional CSEs in terms of N2 amplitude, and the boys had more negative N2 amplitudes in the cI trials than in the iI trials when faced with fearful target expressions in both tasks. Previous research has confirmed that the N2 component is a valid ERP index of cognitive control in children (Rueda et al., 2004; Liu et al., 2015, 2018) and it is related to the implementation of action-monitoring processes, which are related to top-down control to improve subsequent performance and decrease subsequent conflict activation by biasing attention toward task-relevant stimuli (Botvinick et al., 2001; van Veen and Carter, 2002). However, no reliable emotional CSEs were observed in P3 responses among the 5-year-old children in the present study. This pattern of emotional CSEs is contrary to our hypothesis and differs from those identified in previous studies of non-emotional CSEs in preschool children. Some studies found that preschool children’s non-emotional cognitive control was mainly related to P3 but not N2 (Davis et al., 2003; Rueda et al., 2004), while other studies revealed that non-emotional CSEs in 5-year-old children were related to both N2 and P3 (Liu et al., 2018). The present results may be due to the following reason. Davidson et al. (2000) and Davis et al. (2002) noted that when young children must integrate emotional and cognitive processes, two independent but potentially reciprocal subdivisions existed within the ACC, one of which was responsible for cognitive and attentional processes, while the other was responsible for emotional processes, suggesting that the neural mechanism for the integration of emotional and cognitive processes in 5-year-old boys may mainly occur in the ACC and may be related to N2.

Regarding the correlations between the behavioral results and ERP results, we found a significant positive correlation between the RT-CSE values and N2 latency-CSE values in the emotional Flanker task, suggesting that the preschool children’s general processing speed was mainly related to the neural speed of conflict detection. In addition, in the emotional Simon task, the error rate-CSE values were significantly positively correlated to the P3 amplitude-CSE values, which is consistent with the hypothesis that smaller amplitudes of ERP components are associated with better efficiency of conflict resolution (Liu et al., 2018).

### S-S and S-R Emotional Conflict Processing in Young Children

Previous studies of adults have shown that processes on S-S conflicts and S-R conflicts had distinct neural substrates (Fan et al., 2003; Liu et al., 2004; Egner et al., 2007; Li et al., 2017). S-S conflicts activated the inferior frontal cortex, the superior parietal cortex and the right ACC, while S-R conflicts activated the left thalamus and middle frontal cortex, which detect response conflicts and orient spatial attention. The present research examined differences between S-S and S-R conflicts in the emotional CSE context in young children, and the current results are similar to those in a previous study (Liu et al., 2018). The present study found that children had faster responses and shorter N2 latencies in emotional S-S tasks relative to those in S-R tasks, indicating that 5-year-old children have formed different models to process S-S and S-R conflicts in emotional contexts. We further confirmed this result by comparing the CSE values. Specifically, children had smaller RT-CSE values in the emotional Flanker task than those in the Simon task, suggesting that they had better processes in S-S emotional CSEs vs. S-R emotional CSEs. However, these results were modulated by the target facial expression (only the happy target expression), and future studies are needed to confirm this result.

Moreover, an interesting finding was that the 5-year-old children performed better in emotional S-S CSEs than in S-R CSEs only according to the P3 latency-CSE values but not the N2 component. Specifically, the children had smaller P3 latency-CSE values in the emotional Flanker task than in the Simon task, indicating distinctive processing between S-S and S-R emotional CSEs during the conflict resolution stage (related to P3) but not during the emotional conflict monitoring stage. This study provides new evidence confirming that young children may have distinctive neural processes for S-S and S-R conflicts in emotional contexts.

### Different Valences of Facial Expressions and CSEs in Young Children

Pessoa (2009) noted that according to the dual competition model, the cognition resources required for conflict processing were shared by the processing of negative images. Since most studies on the interaction between emotion and CSEs were conducted in adults (e.g., Albert et al., 2010; van Steenbergen et al., 2010; Padmala et al., 2011; Chechko et al., 2014; Fritz et al., 2015; Yang et al., 2016), little is known about the interplay between the identification of different expressions and CSEs during childhood, particularly in young children. Our results revealed that children had faster RTs and lower error rates for happy target expressions than for fearful expressions, which is consistent with previous findings in adults and young children (Chechko et al., 2014; Liu et al., 2017). Chechko et al. (2014) utilized an emotional Stroop task to examine CSEs in adults and revealed that stimuli with happy facial expressions were associated with faster RTs. Liu et al. (2017) also found similar results, with a higher accuracy rate for happy target expressions than that for fearful target expressions in the emotional Flanker task. These findings can be explained by the fact that a happy target expression (low motivational intensity) may broaden the attentional scope in the conflict task, whereas a fearful target expression (high motivational intensity) may narrow the scope (Harmon-Jones et al., 2013). Similarly, van Steenbergen et al. (2010) used a mood induction procedure to trigger negative and positive moods and explored how emotion interacted with CSEs in adults, revealing that the RT-CSE values were increased under the negative mood condition relative to those under the positive mood condition. A later study by Padmala et al. (2011) confirmed these results and also illustrated that negative emotion impaired CSEs in adult samples. The present electrophysiological findings further showed that boys had larger N2 amplitude-CSE values in response to the happy target expressions vs. the fearful target expressions, and the girls exhibited smaller P3 latency-CSE values when faced with the happy target expressions vs. the fearful target expressions in the emotional Simon task. These findings may indicate that the children had better conflict monitoring and resolution processes on happy faces than on fearful faces by activation of frontal (for boys) and parietal functions (for girls).

### Gender Differences in Emotional CSEs in Young Children

In addition, the present study examined the relationship between gender differences and emotional CSEs in 5-year-old preschool children. Similar to previous studies (Boyatzis et al., 1993; Mileva-Seitz et al., 2015), the 5-year-old boys had a higher error rate than the girls on both the emotional Flanker tasks and the emotional Simon tasks, suggesting that young preschool girls may perform better in emotional conflict tasks. By comparing the CSE values, we found that the girls had smaller error rate-CSE values than the boys, and the girls showed larger N2 amplitude-CSE values and smaller P3 latency-CSE values than the boys when faced with fearful target expressions, indicating that the girls’ better performances may be due to their better conflict monitoring and adaptation control processes. These findings are also consistent with those of previous studies showing that preschool girls exhibited better facial processing and emotion identification than boys (Boyatzis et al., 1993; Godard and Fiori, 2010).

### Limitations and Future Work

Several limitations exist in the present study. First, the repetitions of the same face as the target and/or distracter were removed during manipulation of pseudo-randomization. However, the repetitions of the categories of target expressions and the repetitions of some perceptual features (e.g., smiling mouth, etc.) were not strictly controlled and removed, which may induce feature integration biases; therefore, the findings should be interpreted with caution. Second, contingency learning and temporal learning are important theories explaining CSEs. Therefore, in future studies, we should explore whether 5-year-old children display CSEs without feature integration, contingency learning and temporal learning confounds. Third, the remaining numbers of trials were comparatively limited for the ERP analyses because 5-year-old children have higher error rates and exhibit more head movement compared to adults. Their attention spans and motivation to complete the tasks also decreased over the course of the experiment, and in the future, longer break times and a simplified design will be considered.

## Conclusion

Our findings indicated that 5-year-old children exhibited reliable conflict effects and CSEs in the emotional Flanker and Simon tasks. Emotional CSEs can be modulated by the valences of target expressions; specifically, children performed better in CSEs when presented with happy target expressions than with fearful target expressions. Notably, 5-year-old children showed distinct neural processes for S-S and S-R emotional CSEs and performed better on S-S emotional CSEs than on S-R emotional CSEs. In addition, some gender differences in emotional CSEs were observed, with girls outperforming the boys in both conflict detection and conflict resolution. The current study provided a better understanding of how cognition and emotion interact with emotional CSEs among preschool children.

## Author Contributions

DL, TL and JS designed the experiment and wrote the manuscript. DL and TL collected and analyzed the data.

## Conflict of Interest Statement

The authors declare that the research was conducted in the absence of any commercial or financial relationships that could be construed as a potential conflict of interest.
