# Supplementary material for: Conflict Adaptation in 5-Year-Old Preschool Children: Evidence From Emotional Contexts
Source: Front Hum Neurosci. 2019 Jan 29;13:14. doi: 10.3389/fnhum.2019.00014 (PMC6361790; doi:10.3389/fnhum.2019.00014)
Supplement: Supplementary file 1 [file Data_Sheet_1.docx]

**Supplementary files**

**Supplementary File 1—ANOVAs of the congruency effects (conflict effects):**

The mean RTs and error rates as well as the peak latencies and mean amplitudes of N2 and P3 were analyzed using 2×2×2×2×2 ANOVAs with target expression (fearful or happy), task type (emotional Flanker task or emotional Simon task), previous trial (congruent or incongruent) and current trial (congruent or incongruent) as the within-subject variables and gender (boy or girl) as the between-subjects variable.

**Behavioral results:**

Regarding the mean RTs, the main effect of **current trial** was significant, *F*(1,28)=18.41, *p*<0.001, *ŋ2*=0.40, and the children responded more quickly in the congruent trials than the incongruent trials, *t*(29)=4.29, *p*<0.01. The main effect of **previous trial** was also significant, *F*(1,28)=2.90, *p*<0.01, *ŋ2*=0.19. There was a significant main effect of **target expression**, *F*(1,28)=19.79, *p*<0.001, *ŋ2*=0.41. The children displayed longer RTs in response to the fearful target expressions than the happy target expressions, *t*(29)=5.69, *p*<0.001. There was a significant main effect of **task type**, *F*(1,28)=19.79, *p*<0.001, *ŋ2*=0.41. The children displayed longer RTs in the the emotional Simon task than the emotional Flanker task, *t*(29)=4.45, *p*<0.001. There was a significant interaction between **current trial and target expression**, *F*(1,28)=9.51, *p*<0.01, *ŋ2*=0.25, in which the children responded more quickly in the congruent trials than the incongruent trials when faced with happy target expressions, *t* (29)=5.08, *p*＝0.000; moreover, in both the current congruent trials and incongruent trials, the children exhibited longer RTs in response to the fearful target expressions relative to the happy expressions [*t* (29)=7.22, *p*＝0.000, *t* (29)=3.11, *p*＝0.004]. The interaction among **current trial, task type, target expression and gender** was significant, *F*(1,28)=6.42, *p*<0.05, *ŋ2*=0.19; the boys showed longer RTs in the current incongruent trials than current congruent trials when faced with happy target expressions in the emotional Flanker task, *t* (15)=3.68, *p*＝0.023. The interaction among **current trial, task type, target expression and gender** was significant, *F*(1,28)=6.42, *p*<0.05, *ŋ2*=0.19; the boys showed longer RTs in the current incongruent trials than current congruent trials when faced with happy target expressions in the emotional Flanker task, *t* (15)=3.68, *p*＝0.023.

Regarding the mean error rates, there was a significant main effect of **current trial**, *F*(1,28)=4.85, *p*<0.05, *ŋ2*=0.15. The children had higher error rates in the incongruent trials than the congruent trials, *t*(29)=2.09, *p*<0.05. There was a significant main effect of **previous trial**, *F*(1,28)=5.04, *p*<0.05, *ŋ2*=0.15. The main effect of **target expression** was significant, *F*(1,28)=6.98, *p*<0.05, *ŋ2*=0.20, as the children had higher error rates when faced with fearful target expressions than when faced with happy target expressions, *t*(29)=1.94, *p*<0.05. The main effect of **gender** was significant, *F*(1,28)=7.63, *p*<0.05, *ŋ2*=0.21. The boys had higher error rates than the girls, *t*(28)=2.74, *p*<0.05. The interaction among **current trial, target expression and gender** was significant, *F*(1,28)=3.89, *p*<0.05, *ŋ2*=0.12. When faced with happy target expressions, the boys showed higher error rates in the cI trials than the cC trials, *t*(15)=4.13, *p*<0.05; the boys had higher error rates than the girls in the cI trials when faced with both fearful and happy target expressions, *t*(15)=3.15, *p*<0.05, and *t*(15)=2.03, *p*<0.05. There was a significant interaction between **current trial and target expression**, and the children showed higher error rates when faced with fearful target expressions relative to happy expressions, *t*(29)=3.27, *p*=0.003; moreover, the children had higher error rates in the incongruent trials relative to the congruent trials when faced with happy target expressions, *t*(29)=3.26, *p*=0.002. The **current trial × target expression × task type** interaction was significant, *F*(1,28)=10.95, *p*<0.05, *ŋ2*=0.28; when faced with happy target expressions, the participants showed higher error rates in the incongruent trials than the congruent trials in the emotional Flanker task, *t*(29)=4.12, *p*=0.000.

**ERP results**

**N2:**

Regarding the means of the N2 mean amplitudes, the main effect of **task type** was significant, *F*(1,28)=7.02, *p*<0.05, *ŋ2*=0.20, as the children exhibited more negative N2 components in the emotional Simon task than the emotional Flanker task, *t*(29)=2.65, *p*<0.05.

Regarding the means of the N2 peak latencies, there was a significant main effect of **previous trial**, *F*(1,28)=5.85, *p*<0.05, ŋ2=0.17, as the children showed longer N2 latencies in the incongruent trials than congruent trials, *t*(28)=2.42, *p*<0.05. There was a significant main effect of **current trial**, *F*(1,28)=3.48, *p*<0.05, ŋ2=0.11, as the N2 latencies in the incongruent trials were longer than those in the congruent trials, *t*(28)=1.86, *p*<0.05. The main effect of **task type** was significant, *F*(1,28)=32.28, *p*<0.001, *ŋ2*=0.54. The post hoc tests revealed that the N2 latencies in the emotional Flanker task were shorter than those in the emotional Simon task, *t*(28)=5.68, *p*<0.001.

**P3:**

Regarding the means of the P3 mean amplitudes, the main effect of **target expression** was significant, *F*(1,28)=9.59, *p*<0.01, *ŋ2*=0.26. The children showed larger P3 amplitudes in response to the fearful target expressions relative to the happy target expressions, *t*(29)=2.68, *p*<0.01. There was a 4-way interaction among **current trial, target expression, task type and gender,** *F*(1,28)=7.79, *p*<0.01, *ŋ2*=0.22; in the emotional Simon task, the boys showed larger P3 amplitudes in the incongruent trials relative to the congruent trials when faced with happy target expressions, *t*(29)=2.81, *p*=0.009; when faced with happy target expressions, the boys exhibited larger P3 amplitudes in the emotional Simon task than the emotional Flanker task in the incongruent trials, *t*(13)=3.85, *p*=0.001. When faced with happy target expressions, in the congruent trials of the emotional Flanker task, the boys exhibited larger P3 amplitudes than the girls [*t*(28)=2.35, *p*=0.025].

Regarding the means of the P3 mean latencies, the main effect of **current trial** was significant, *F*(1,28)=4.02, *p*<0.05, *ŋ2*=0.13, as longer P3 latencies were found in the incongruent trials than congruent trials, *t*(29)=1.43, *p*<0.05.

**Supplementary Figure 1—Globally averaged N2 and early P3 waveforms of congruency effects (conflict effects)**

**Supplementary Figure 1.** Globally averaged N2 and early P3 waveforms of conflict effects

**
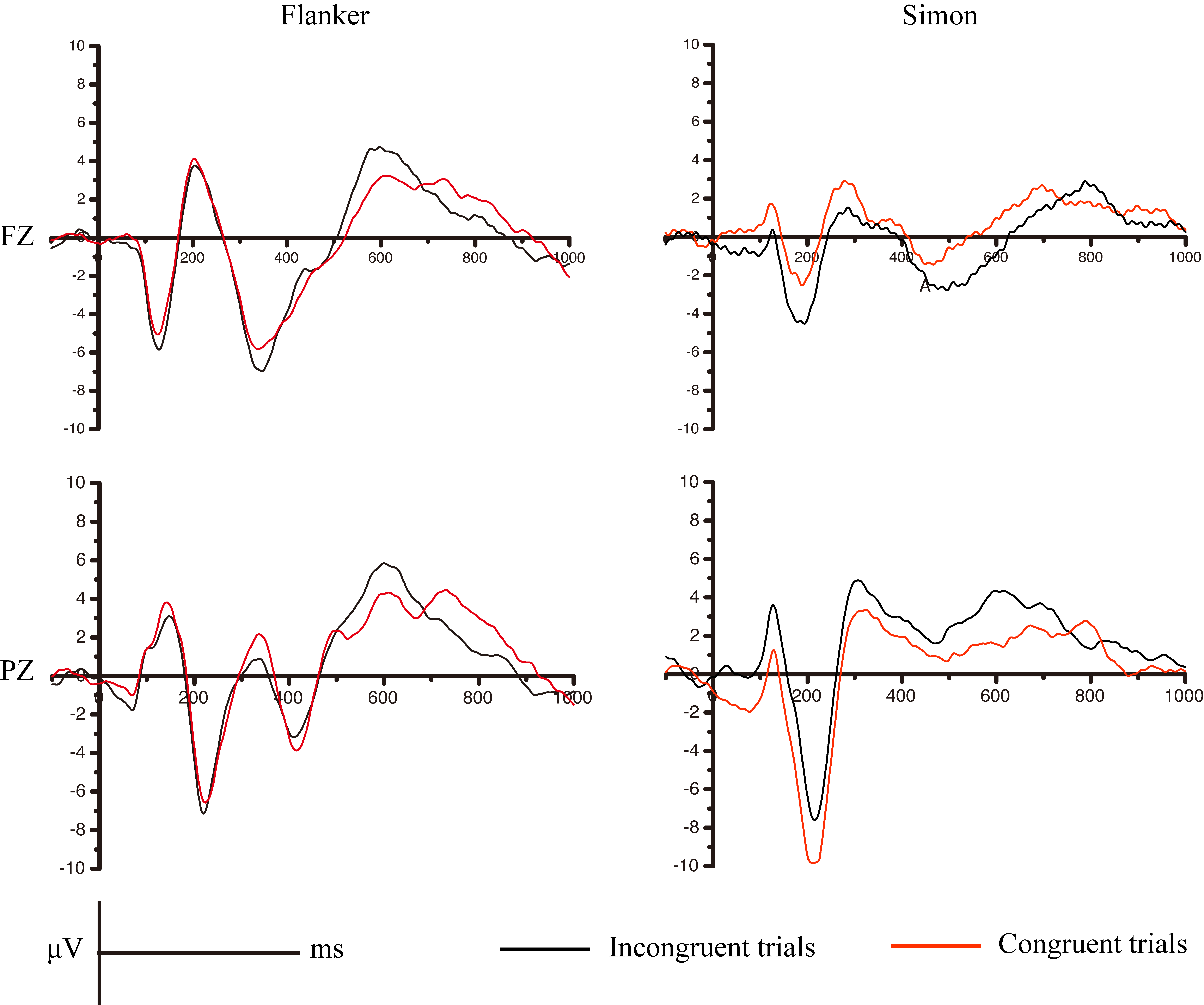
**
